# Simulated Climate Change Enhances Microbial Drought Resilience in Ethiopian Croplands but Not Forests

**DOI:** 10.1111/gcb.70065

**Published:** 2025-03-05

**Authors:** Lettice C. Hicks, Ainara Leizeaga, Carla Cruz Paredes, Albert C. Brangarí, Dániel Tájmel, Menale Wondie, Hans Sandén, Johannes Rousk

**Affiliations:** ^1^ Microbial Ecology, Department of Biology Lund University Lund Sweden; ^2^ Amhara Agricultural Research Institute (ARARI) Bahir Dar Ethiopia; ^3^ Forest Ecology, Department of Forest and Soil Sciences University of Natural Resources and Life Sciences (BOKU) Vienna Austria

**Keywords:** climate change, climate warming, drought, land‐use change, microbial community structure and function, soil carbon cycling, tropical ecosystems

## Abstract

Climate change and land‐use change represent a dual threat to terrestrial ecosystem functioning. In the tropics, forest conversion to agriculture is occurring alongside warming and more pronounced periods of drought. Rainfall after drought induces enormous dynamics in microbial growth (potential soil carbon storage) and respiration (determining carbon loss), affecting the ecosystem carbon budget. We investigated how legacies of drought and warming affected microbial functional (growth and respiration) and structural (16S and ITS amplicon) responses after drought. Rain shelters and open‐top chambers (OTCs) were used to simulate drought and warming in tropical cropland and forest sites in Ethiopia. Rain shelters reduced soil moisture by up to 25 vol%, with a bigger effect in the forest, while OTCs increased soil temperature by up to 6°C in the cropland and also reduced soil moisture but had no clear effect in the forest. Soils from these field treatments were then exposed to a standardized drought cycle to test how microbial community traits had been shaped by the different climate legacies. Microbial growth started increasing immediately after rewetting in all soils, reflecting a resilient response and indicating that microbial communities perceived the perturbation as relatively mild. Fungi recovered faster than bacteria, and the recovery of fungal growth was generally accelerated in soils with a legacy of drought. Microbial community functions and structures were both more responsive in the cropland than in forest soils, and a legacy of drought particularly enhanced microbial growth and respiration responses in the cropland but not the forest. Microbial communities in cropland soils also used carbon with a higher efficiency after rewetting. Together, these results suggest contrasting feedbacks to climate change determined by land use, where croplands will be associated with mitigated losses of soil carbon by microorganisms in response to future cycles of drought, compared to forests where soil carbon reservoirs remain more sensitive.

## Graphical abstract

Rainfall after drought induces enormous dynamics in microbial growth (potential soil carbon storage) and respiration (determining carbon loss), affecting the ecosystem carbon budget. We investigated how legacies of drought and warming affected microbial functional and structural responses after drought, using soils from tropical cropland and forest sites in Ethiopia. Our results suggest contrasting feedbacks to climate change determined by land use, where croplands will be associated with mitigated losses of soil carbon by microorganisms in response to future cycles of drought, compared to forests where soil carbon reservoirs remain more sensitive.

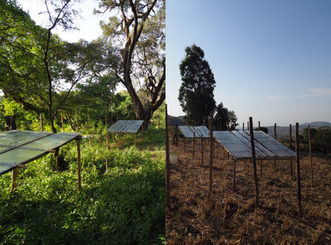

## 1 Introduction

Climate change is occurring rapidly across the globe, with warming and an intensification of hydrological regimes resulting in more pronounced periods of drought followed by heavy rainfall events (IPCC 2023; Yuan et al. 2023). Tropical ecosystems make a quantitatively important contribution to the global carbon (C) budget, with tropical forests accounting for more than two‐thirds of terrestrial plant biomass and storing around one‐third of all soil C, despite covering only 15% of land area (Jobbágy and Jackson 2000; Pan et al. 2013; Jackson et al. 2017). While there is an urgent need to understand the response of terrestrial ecosystems to future climate change, tropical regions remain understudied in general, with a specific lack of information from drier regions receiving < 1500 mm annual rainfall (Cavaleri et al. 2015). In conjunction with climate change, changes in land use in tropical ecosystems, including the conversion of forests to croplands and agricultural intensification, are also occurring (Lambin, Geist, and Lepers 2003; Don, Schumacher, and Freibauer 2011). Identifying how drought and warming will interact with land use to affect future soil C dynamics is therefore vital in order to understand the biological feedback to climate change.

Rewetting of dry soil after a period of drought induces pronounced changes in microbial growth and biogeochemistry, including a pulse of CO_2_ release to the atmosphere (Birch 1958; Jarvis et al. 2007). During this period of high CO_2_ release, soil microorganisms have been found to exhibit different growth responses, ranging from (1) a more resilient response where growth starts increasing immediately and linearly upon rewetting to rapidly recover to pre‐disturbance levels to (2) a more sensitive response where there is a pronounced lag period of near‐zero growth before microbial growth starts to increase exponentially, only recovering to or surpassing rates in non‐perturbed soils after a delay of up to 30 h (Göransson et al. 2013; Meisner, Bååth, and Rousk 2013; Meisner, Rousk, and Bååth 2015). The time taken for microbial growth to recover after drought will have a large impact on the ecosystem C budget (Rousk and Brangarí 2022), since low growth during a period of high respiration (i.e., low microbial C use efficiency; CUE) will result in more C lost to the atmosphere relative to that retained by microorganisms in the soil (Liang, Schimel, and Jastrow 2017; Camenzind et al. 2023).

The type of microbial response after drought is thought to be linked to the “harshness” of the disturbance as perceived by microorganisms (Meisner et al. 2017), which can be affected by both the severity of the actual disturbance as well as historical exposure to drying‐rewetting cycles (“legacy effect”; Hawkes and Keitt 2015). Legacies of drought—simulated using rain‐exclusion shelters—have been shown to accelerate the recovery of bacterial growth (de Nijs et al. 2019), as well as reduce the respiration release from soils after drying‐rewetting (Veach and Zeglin 2020). A legacy of drought may therefore select for a microbial community able to recover their growth rates more quickly and efficiently after a drought cycle. Indeed, studies across natural precipitation gradients of both arid (Leizeaga et al. 2021) and humid (Tang et al. 2023) ecosystem types found that microorganisms from historically drier sites exhibited a higher CUE following rewetting than those from historically wetter sites. A legacy of drought could also impact microbial process rates under steady‐state conditions (i.e., stable rates at optimal moisture). For example, reduced inputs of plant‐derived C during the drought period (Ruehr et al. 2009; Fuchslueger et al. 2014) could lead to lower rates of microbial growth and respiration in soils after the drought has ended (Hicks et al. 2018), further contributing to a climate legacy.

Some studies have also observed differences in microbial responses to drying‐rewetting depending on land use. For example, more pronounced respiration responses were measured in soils from an oak forest compared to an adjacent grassland (Fierer and Schimel 2002), while microbial growth after rewetting was more resilient in cropland than pasture soils (Brangarí, Lyonnard, and Rousk 2022). Land use may influence the susceptibility of soils to drying (Olorunfemi and Fasinmirin 2017), as well as resource availability to microorganisms (Singh et al. 2020), thereby modulating microbial responses to drought. Soil temperature and moisture in forests and croplands typically differ within the same climatic zone, as forest soils are usually cooler and moister due to the litter layer and canopy shading, which buffers soils against large changes in temperature and moisture (Savva et al. 2010). Plants in forests also often have deeper roots, which may help to lift and redistribute water from deeper in the soil profile to the surface (Horton and Hart 1998). In contrast, the soil surface in croplands is more exposed, meaning that during dry periods, cropland soils may dry faster and experience more severe moisture deficit compared to forest soils. In addition, different plant communities may contribute to differences in the quantity and quality of SOM (Woloszczyk et al. 2020). In soils where resource availability is higher, microbial communities may be able to allocate more C to e.g., osmolyte production in order to better resist the physiological stress associated with drought (Schimel et al. 2007; Malik and Bouskill 2022). More frequent physical disturbances in croplands, e.g., due to ploughing, may also affect resource availability (Reinsch et al. 2018) and/or select for a microbial community more resilient to disturbances (Philippot, Griffiths, and Langenheder 2021).

After a drought, changes in abiotic conditions may trigger a succession of microbial community assembly. During dry‐down, the reduction in water may favor more drought‐tolerant taxa associated with a *K*‐selected life strategy (de Vries and Shade 2013). For example, one study of grassland soils found an increased relative abundance of Actinobacteria during drying (Barnard, Osborne, and Firestone 2013), with taxa from this phylum found to be particularly tolerant to low moisture levels (Goodfellow and Williams 1983; Zvyagintsev et al. 2007). In contrast, the dramatic increase in resource availability associated with rewetting after drought (Slessarev et al. 2020) may select for faster‐growing *r*‐strategists, who are able to outcompete the *K*‐selected taxa under high resource supply. Consistent with this, another study in a Californian grassland found that the initial bacterial growth response after rewetting was dominated by Proteobacteria (especially Burkholderiales) and Firmicutes (especially Bacillales) (Blazewicz et al. 2020), with these taxa associated with fast growth rates under high C availability (Morrissey et al. 2019). Over time, as resources become depleted, *K*‐selected taxa may again outcompete *r*‐strategists, contributing to a systematic shift in microbial community structure following the disturbance. However, an open question remains in soil ecology regarding how changes in microbial taxa correspond to changes in community‐level functioning (Foley et al. 2024; Lennon et al. 2024).

Here we investigated how climate legacies of drought and warming affected microbial functional and structural responses to rewetting after a period of drought and how these responses interacted with the land use. To do so, we used soils from climate manipulation experiments in Ethiopia, where rain shelters were used to simulate drought and open‐top chambers (OTCs) were used to simulate warming. These field treatments were applied in both a degraded cropland and a pristine forest, enabling comparison between the two landuses. Soils were sampled 1.5 years after the field treatments had been established, with these soils then subjected to a standardized drought‐cycle in the laboratory in order to assess how microbial community traits had been shaped by the different climate legacies. Our aim was to assess the legacy effect of simulated drought and warming on microbial responses after drought and to test how differences in microbial community structure were linked to function during this time. We hypothesized that the different climate legacies and land uses would influence microbial process rates and responses after drought. Specifically, we hypothesized (i) that a legacy of drought (i.e., rain shelter treatments) would result in lower microbial process rates under steady‐state conditions, but more resilient and efficient microbial responses to the experimental drought‐cycle; (ii) that microbial responses to drying‐rewetting would be more resilient, and would occur with a higher CUE, in the cropland than forest soils, due to the history of higher exposure to drying‐rewetting cycles in weather‐exposed croplands, and finally, (iii) that differences in microbial resilience after drought would be associated with initial differences in microbial community structures, and that there would be an ordered succession of microbial community responses over time, with these changes in microbial community structure linked to changes in function.

## 2 Materials and Methods

### 2.1 Site Description

The studied cropland and forest sites are located in the northwestern Ethiopian Amhara region at 2110 m elevation and ca. 4 km apart (Tara Gedam; 12°9′42″ N, 37°44′2″ E and 12°8′42″ N, 37°44′35″ E, respectively). The chosen sites were selected from previously studied sites in the Amhara National Regional State (Leizeaga et al. 2022). The area is characterized by a mean annual temperature (MAT) of 19°C and mean annual precipitation (MAP) of 1100 mm year^−1^. The majority of rainfall occurs between June and September, with a little rain in April and May, and the period between October and March being generally dry. The soils are classified as Cambisols, with a clayey to silty texture and < 10% sand (Assefa et al. 2017).

Two contrasting land uses were selected for the study. The forest site was a dry Afromontane remnant pristine forest, comprised of indigenous tree species, which is protected by local institutions associated with churches and monasteries (Aerts et al. 2016). In the cropland site, the soil is ox‐ploughed to a tillage depth of 10–20 cm, and crop rotations include Teff ( *Eragrostis tef* ), Finger millet ( *Eleusine coracana* ), Sorghum ( *Sorghum bicolor* ), Maize ( *Zea mays* ), Bread wheat ( *Triticum aestivum* ), Niger seed (*Guizotiaabyssinica*), and Faba bean ( *Vicia faba* ).

### 2.2 Field Experiment and Monitoring

Field experiments were established in November 2017 in both forest and cropland sites. At each site, a 12 m × 6 m area was fenced, within which four drought and three warming plots were established, along with corresponding control plots in a randomized blocked design. Drought was simulated with the use of rain‐exclusion shelters. A 1.8 m × 1.8 m area was selected, above which a rain shelter was built. Four eucalyptus wooden beams were used to support the structure, with two of them being 1.5 m high and the other two 1 m to form an incline such that water would flow in the same direction as the slope in the site, thereby running away from the plot. These beams were topped with a square wooden frame to which a transparent plastic cover was secured, covering 80% of the area, thereby reducing 80% of the incoming rainfall (hereafter “drought treatment”). Equivalent 1.8 m × 1.8 m plots served as a control. OTCs were installed to passively increase the air temperature (hereafter “warming treatment”). These OTCs were built according to the International Tundra Experiment (ITEX) design (Marion et al. 1997), with the OTCs being 3 mm thick and 35 cm tall, with an 85 cm diameter at the top and a 150 cm diameter at the base. The fenced areas where the field experiments were established were maintained and monitored weekly. Vegetation was continuously removed by weekly mowing in both the cropland and the forest to compensate for the lack of grazing, thus ensuring a similar vegetation before and after the fencing.

**FIGURE 1:**
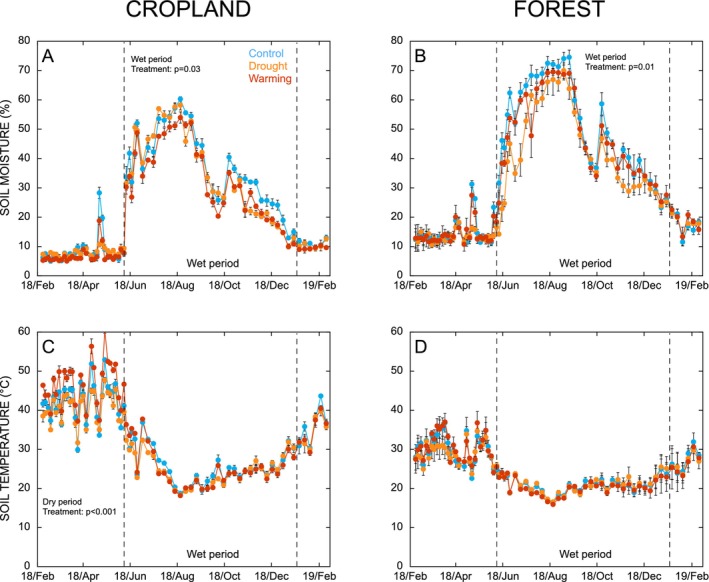
Volumetric soil moisture (%) (Panels A, B) and temperature (°C) (Panels C, D) in cropland and forest sites. Data points denote mean ± SE (*n* = 4 for control and drought treatments and *n* = 3 for warming treatment). Vertical dashed lines indicate the transition between dry and wet periods.

Soil volumetric water content (vol%) and temperature (°C) were monitored in all plots twice weekly during the course of the field experiment (1.5 years) using moisture and temperature probes (SM150T, Delta‐T Devices, which measures soil volumetric moisture between 0 and 5 cm depth and a digital thermometer with a stainless‐steel sensor marked at 5 cm depth). Soil temperature and moisture varied seasonally, with soils generally being cooler and wetter between June and February (hereafter referred to as “wet period”; Figure 1). During the dry period, soil moisture was consistently low, irrespective of field treatments. However, during the wet period, there was a significant effect of the field treatments on soil moisture in both the forest (*p* = 0.01) and cropland (*p* = 0.03). In the forest, the maximum effect occurred at the start of the wet period, in early June, where soil moisture was 26.4 ± 3.5 vol% lower in the drought treatment than the control (Figure 1B). In the cropland, the maximum effect occurred towards the end of the wet period, in late October, where soil moisture was 10.4 ± 0.4 vol% and 9.6 ± 5.0 vol% lower in the drought and warming treatments than the control, respectively (Figure 1A). In the cropland there was also a significant effect of the field treatments on soil temperature during the dry period (*p* < 0.001). In this case, the maximum effect occurred towards the end of the dry period, in late May, when soil temperature was 6.5°C ± 0.5°C higher in the warming treatment than in the control (Figure 1C). There was no clear effect of the field treatments on soil temperature in the forest (Figure 1D).

### 2.3 Soil Sampling

Soils were sampled towards the end of the dry season in February 2019 when soil moisture was low (Figure 1). Within each plot, five soil samples (0–5 cm depth) were collected to form a composite sample, excluding the area within a 10 cm perimeter of the rain‐exclusion shelters and OTCs to avoid edge effects. At both the cropland and forest sites, four control samples, four drought treatment samples and three warming treatment samples, were collected, resulting in a total of 11 samples for each land use. After sampling, the soils were stored in double ziploc bags in cooled, insulated boxes (ca. 5°C) and were express shipped to Lund University, Sweden, where samples were stored at 4°C until the start of the experiment within 2 weeks.

### 2.4 Soil Characterization

All field‐moist soil samples were sieved (< 4 mm), and stones and roots were manually removed. Soil pH and electrical conductivity (EC) were measured in a 1:5 (w:V) water extraction. Soil subsamples were used to measure gravimetric soil moisture (105°C overnight) and soil organic matter content (SOM) by loss on ignition (600°C for 12 h). Maximum water holding capacity (WHC) was determined as described by Hicks et al. (2018).

### 2.5 Drying‐Rewetting Experiment

To assess how microbial community traits had been shaped by different climate legacies, all soils were exposed to a standardized drought cycle (Figure S1). To do so, soil samples were air‐dried under a fan at room temperature for 3 days, reaching stable air‐dry conditions of no detectable microbial activity (corresponding to a water potential of ca. −100 MPa). 1.0 g subsamples of air‐dried soils were weighed into different tubes, with these soils then rewetted to an optimal moisture content for microbial activity (50% WHC corresponding to ca. −30 kPa) using distilled water. After rewetting, the soils were incubated at 17°C (corresponding to the mean annual temperature of the sites; Leizeaga et al. 2022) for up to 3 weeks, during which bacterial growth, fungal growth, and respiration (see below) were measured at high temporal resolution by destructive harvesting of the soils in different tubes over time. To facilitate this, samples were rewetted in two sets, with one set rewetted in the morning and the other in the evening, with these two sets monitored in parallel and data subsequently combined to generate one time series (Meisner, Bååth, and Rousk 2013; Meisner, Rousk, and Bååth 2015). For measurements of bacterial and fungal growth and respiration, samples were assessed at 3, 9, 15, 21, 31, 42, 60, 84, 132, 248, 271, 295, 319, 367, 415, 439, 463, and 487 h after rewetting. Soil subsamples were also used to characterize the microbial community, with samples taken before rewetting (i.e., in air‐dry soils), as well as 24, 72, 170, and 470 h after rewetting.

### 2.6 Microbial Growth, Respiration, and Carbon Use Efficiency

Bacterial growth was determined by measuring the rate of ^3^H‐leucine (Leu) incorporation into extracted bacteria (Bååth, Pettersson, and Söderberg 2001; Rousk, Brookes, and Bååth 2009). To do so, 1.0 g of soil was mixed with 20 mL of demineralized water by vortexing for 3 min and then centrifuging (10 min at 1000 *g*). The resulting bacterial suspension was incubated for 1 h with 2 μL 1‐[4,5‐^3^H]‐Leucine (5.7 TBq mmol^−1^, Perkin Elmer, USA) and unlabelled Leu with a final concentration of 275 nM Leu in the bacterial suspension. Bacterial growth was then terminated by adding 75 μL of 100% trichloroacetic acid. Centrifugation and washing were performed as described by Bååth, Pettersson, and Söderberg (2001). The amount of leucine incorporated into extracted bacteria was used as a measure of bacterial growth. In the same soils, we also measured the rate of ^3^H‐thymidine (TdR) incorporation into DNA, enabling bacterial growth rates to be converted to bacterial C production (μg C g^−1^ SOM h^−1^) according to Soares and Rousk (2019).

Fungal growth was determined by using the ^14^C‐acetate‐in‐ergosterol method (Newell and Fallon 1991) adapted for soil (Rousk, Brookes, and Bååth 2009). To do so, 1.0 g of soil was mixed with 20 μL of ^14^C‐acetate solution ([1‐^14^C] acetic acid, sodium salt, 2.07 GBq mmol^−1^, Perkin Elmer) and unlabelled sodium acetate, resulting in a final acetate concentration of 220 μM in the soil slurry. Samples were incubated for 2 h before 10% formaldehyde was added to terminate growth. Ergosterol and incorporated acetate were measured according to Rousk and Bååth (2007). The rate of acetate incorporation into ergosterol was converted to fungal‐C production (μg C g^−1^ SOM h^−1^) according to Soares and Rousk (2019).

To measure respiration, 1.0 g of soil was weighed into 20 mL glass vials. The vials were then purged with pressurized air and sealed with crimp caps before incubating for between 6 and 72 h (with a shorter incubation time at the start of the experiment) in the dark at 17°C. The CO_2_ production was measured using a gas chromatograph equipped with a methanizer and a flame ionization detector.

Microbial carbon use efficiency (CUE) was estimated as the ratio of the total microbial C production (bacterial growth + fungal growth) to the total microbial carbon use (total microbial growth + respiration). The ratio between fungal growth and bacterial growth (both in units of C) was also calculated.

### 2.7 Microbial Community Characterization

DNA was extracted from 250 mg of freeze‐dried soil using MoBio Power Soil Kits (MoBio, Carlsbad, CA, USA) following the manufacturer's instructions. DNA was quantified fluorometrically (Qubit, Invitrogen, Carlsbad, CA, USA), and DNA extracts were sent to BGI for amplicon sequencing. For bacterial communities, the V3‐V4 region of the 16S was amplified using the primers 341F (5′‐CCTAYGGGRBGCASCAG‐3′) and 806R (5′‐GGACTACNNGGGTATCTAAT‐3′). For fungal communities, the ITS1‐ITS2 region was amplified using the primers ITS1 (5′‐CTTGGTCATTTAGAGGAAGTAA‐3′) and ITS2 (5′‐GCTGCGTTCTTCATCGATGC‐3′).

### 2.8 Data Analysis

#### 2.8.1 Microbial Growth, Respiration and CUE Responses to Drying‐Rewetting

Cumulative microbial growth (bacterial growth + fungal growth) and respiration, along with CUE and the ratio of fungal‐to‐bacterial growth, were calculated during 1 day, 1 week, and 3 weeks following the experimental drought cycle. As the effect of the field treatments on soil temperature and moisture differed between land uses (Figure 1), it was necessary to evaluate the effects of the field‐treatments on microbial functional and structural responses separately by land use. The effect of field treatments on the cumulative values of microbial growth, respiration, CUE, and fungal‐to‐bacterial growth ratio in forest and cropland soils was tested using a mixed effect model, where field treatment (control, drought, and warming) was included as a fixed effect and plot nested within block was included as a random effect. Pairwise comparisons of significant effects were assessed by Tukey's post hoc tests, with significant differences identified where *p* < 0.05.

To evaluate the rate of microbial recovery after the disturbance, we calculated the time taken for bacterial growth, fungal growth, and microbial CUE to return to the steady‐state level. To do so, the steady‐state level was determined as the average between 250 h and 500 h after rewetting (i.e., when growth rates and CUE had reached a steady‐state; see Section 3). Then, a linear model was fitted to the increase in bacterial growth, fungal growth, and CUE over time, from which the time taken to reach the steady‐state level was determined. The effect of field treatment on steady‐state microbial rates and recovery times was tested using mixed models, as previously described. The recovery times were also regressed against the soil moisture of the air‐dried soils to test whether small variations in moisture prior to rewetting affected the time for microbial recovery. Statistics were performed using JMP Pro 16 (SAS institute).

#### 2.8.2 Soil Microbial Communities

All sequence data was processed using DADA2 (Callahan et al. 2016) to determine the amplicon sequence variants (ASVs). For the bacterial community analysis, the forward (200 bp) and reverse reads (160 bp) were trimmed and merged. For fungi, the DADA2 ITS Pipeline Workflow (1.8) was used with default parameters. The databases SILVA (Quast et al. 2012) for bacteria and UNITE (Abarenkov et al. 2024) for fungi were used for taxonomic identification of the ASVs. To assess how microbial communities had been affected by landuse and field treatments, bacterial and fungal alpha and beta diversity from the air‐dried soils (i.e., before rewetting) were estimated. The shannon diversity index was calculated to estimate alpha diversity. For beta diversity, ASVs were filtered by only keeping ASVs with at least 5 counts before samples were transformed to even sampling depth using the function *transform_sample_counts* from the *phyloseq* package (McMurdie and Holmes 2013). Principal coordinate analysis (PCoA) based on Bray‐Curtis dissimilarities was used to visualize the beta diversity, plotting both the taxa and the samples, separately by land use. PERMANOVA was performed to test if there were differences in beta diversity between field‐treatments. We also assessed changes in bacterial and fungal community structures over the course of the disturbance, separately by land‐use. The datasets were filtered by removing ASVs that did not appear more than five times in more than half the samples for bacteria and in more than one fifth of the samples for fungi. These datasets were then used to calculate beta diversity with these data used for further analyses. To assess if initial structural differences could explain differences in bacterial and fungal resilience to the experimental drought cycle, regression analyses of the bacterial and fungal recovery times and PCoA scores were conducted. We also tested whether changes in cumulative bacterial and fungal growth over time were linked to the observed changes in microbial community structures after rewetting. Microbial community analyses were performed using R version 4.2.2 (R Core Team 2022).

## 3 Results

### 3.1 Soil and Microbial Community Characterization

There was generally no effect of the field treatments on soil physicochemical characteristics (pH, EC, SOM, and WHC) in either the cropland or forest sites (Table 1). This was with the exception of soil pH in the cropland, which was marginally lower in the drought treatment than the control and warming treatments (*p* = 0.05; Table 1).

**TABLE 1:** Soil characteristics.

Land use	Treatment	pH	EC (μS cm^−1^)	SOM (%)	Maximum WHC (g H_2_O g^−1^ dwt)
Cropland	Control	6.8 ± 0.03	46 ± 1	8.8 ± 0.2	0.71 ± 0.01
	Drought	6.4 ± 0.03	52 ± 4	9.3 ± 0.4	0.72 ± 0.04
	Warming	6.7 ± 0.21	46 ± 5	8.6 ± 0.1	0.70 ± 0.02
Forest	Control	6.7 ± 0.03	66 ± 3	18.9 ± 0.8	0.85 ± 0.01
	Drought	6.7 ± 0.04	71 ± 4	19.5 ± 1.5	0.84 ± 0.03
	Warming	6.7 ± 0.06	61 ± 6	18.5 ± 0.7	0.80 ± 0.02

*Note:* Data represents mean ± SE (*n* = 4 in control and drought treatments and *n* = 3 in warming treatment).

Abbreviations: EC, electrical conductivity; SOM, soil organic matter; WHC, water holding capacity.

**FIGURE 2:**
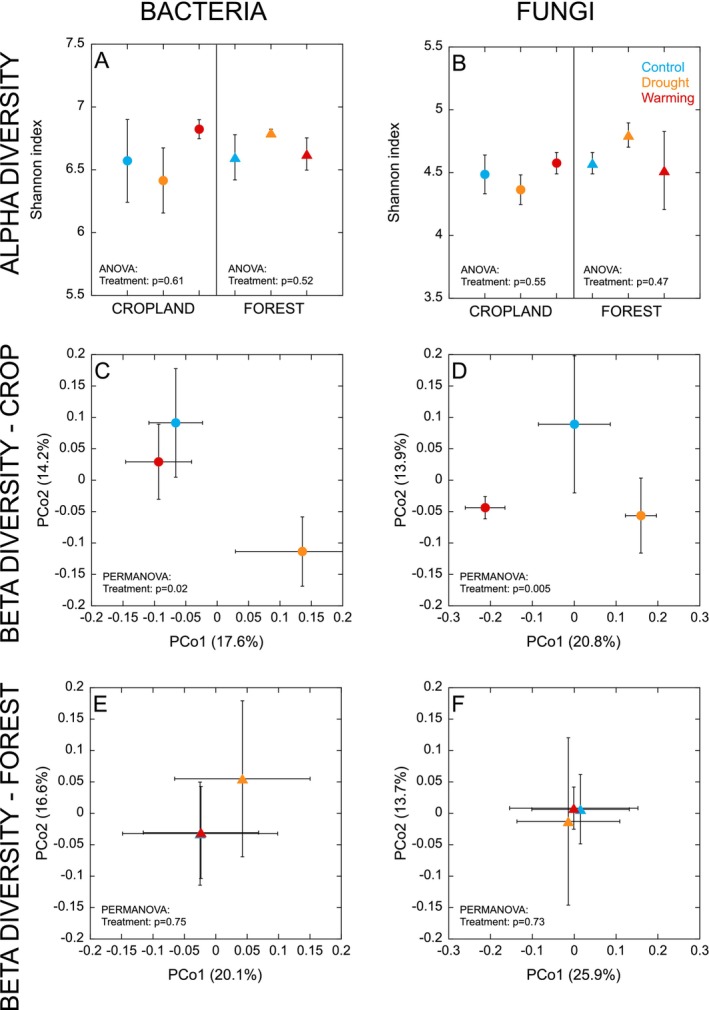
Bacterial (Panels A, C, E) and fungal (Panels B, D, F) alpha (Shannon index) and beta diversity measured in air‐dry soils from cropland and forest sites. Data represent mean ± SE (*n* = 4 for control and drought treatments and *n* = 3 for warming treatment; where error bar cannot be seen, the bar is smaller than the symbol). Phyla explaining variation in the initial bacterial and fungal community structures (Panels C–F) are provided in Figure S2.

The alpha diversity (Shannon index) of bacteria and fungi was unaffected by field treatments in both land uses (all *p* > 0.47; Figure 2A,B). However, in the cropland, there was a significant effect of field treatments on bacterial (*p* = 0.02) and fungal (*p* = 0.005) beta diversity. In this case, the bacterial community structure in soils from the drought treatment differed from that in the control and warming treatments (Figure 2C), with these differences associated with a higher relative abundance of Planctomycetes, Fibrobacteres, and Deinococcus‐Thermus in the drought treatment and a higher relative abundance of Chlamydiae and Chloroflexi in the control and warming treatments (Figure S2A). The fungal community structure in the cropland also differed between the drought and warming treatments, with the control intermediate (Figure 2D). The drought treatment was associated with a higher relative abundance of Aphelidomycota, and the warming treatment was associated with a higher relative abundance of Rozellomycota, Entorrhizomycota, and Mortierellomycota, compared to the control, which was associated with a higher relative abundance of Glomeromycota and Basidomycota (Figure S2B). There was no effect of the field treatments on bacterial (*p* = 0.75) or fungal (*p* = 0.73) community structures in the forest (Figure 2E,F).

### 3.2 Microbial Growth and Respiration Responses to Drying‐Rewetting

**FIGURE 3:**
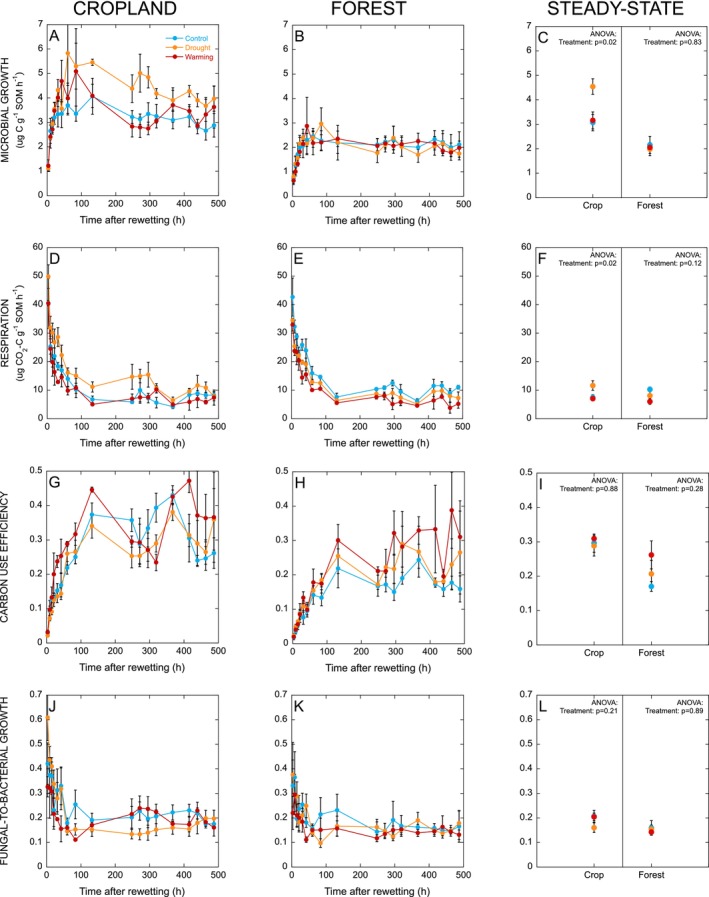
Microbial growth (Panels A–C), respiration (Panels D–F), carbon use efficiency (Panels G–I), and the ratio of fungal‐to‐bacterial growth (Panels J–L) in cropland and forest soils after drying‐rewetting and at steady‐state (see Section 2.8). Data represent mean ± SE (*n* = 4 for control and drought treatments and *n* = 3 for warming treatment).

The response of all measured microbial processes following rewetting was similar in both land uses and all field treatments, with microbial growth rates starting to increase immediately after rewetting (Figure 3A,B). The microbial response in the cropland soil was more dynamic, with growth rates initially exceeding the steady‐state level to reach a peak ca. 60 h after rewetting, followed by a gradual decline towards a stable level. In contrast, in the forest soil, growth rates simply increased to reach a steady state which was then maintained. In the cropland, there was a significant effect of field treatment on the steady‐state rate of microbial growth (*p* = 0.02), with higher rates of growth in soil from the drought treatment than the control and warming treatments (Figure 3C). In the forest soils, field treatments had no effect on steady‐state rates of microbial growth (*p* = 0.83).

In all cases, respiration peaked immediately after rewetting, followed by an exponential decrease towards the steady‐state rate, which was reached after approximately 1 week (Figure 3D,E). Like for microbial growth, there was a significant effect of field treatment on the steady‐state respiration rate in the cropland (*p* = 0.02), with higher rates in soils from the drought treatment, but no significant effect of field treatment on steady‐state respiration in the forest (Figure 3F).

Microbial CUE was initially very low but started increasing immediately after rewetting, reaching steady‐state values of ca. 0.20–0.30 by the end of the first week (Figure 3G,H). There was no significant effect of field treatments on the steady‐state CUE in either the cropland or forest soils (Figure 3I). The ratio of fungal‐to‐bacterial growth decreased rapidly during the first 24 h after rewetting and then stabilized at a similar level (ca. 0.15) in all soils (Figure 3J–L). There was no significant effect of field treatments on the steady‐state ratio of fungal‐to‐bacterial growth in either the cropland or forest soils (Figure 3L).

**FIGURE 4:**
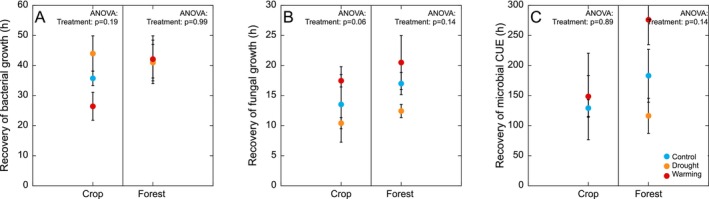
Recovery time (hours) for bacterial growth rates (Panel A), fungal growth rates (Panel B), and microbial CUE (Panel C) after drying‐rewetting (see Section 2.8). Data represent mean ± SE (*n* = 4 for control and drought treatments and *n* = 3 for warming treatment).

Fungal growth (15 ± 1 h) recovered more quickly than bacterial growth (39 ± 3 h) after rewetting (*p* < 0.001; Figure 4A,B). In both land uses there was no significant effect of field treatments on the recovery time for bacterial growth, fungal growth, or CUE (Figure 4A–C). However, there was a tendency for a faster recovery of fungal growth in both the cropland and forest soils with a legacy of drought (Figure 4B). There was no significant relationship between the moisture content of the air‐dry soils prior to rewetting and the recovery time for bacterial growth (cropland *p* = 0.78 and forest *p* = 0.27), fungal growth (cropland *p* = 0.92 and forest *p* = 0.69), or CUE (cropland *p* = 0.70 and forest *p* = 0.26).

**FIGURE 5:**
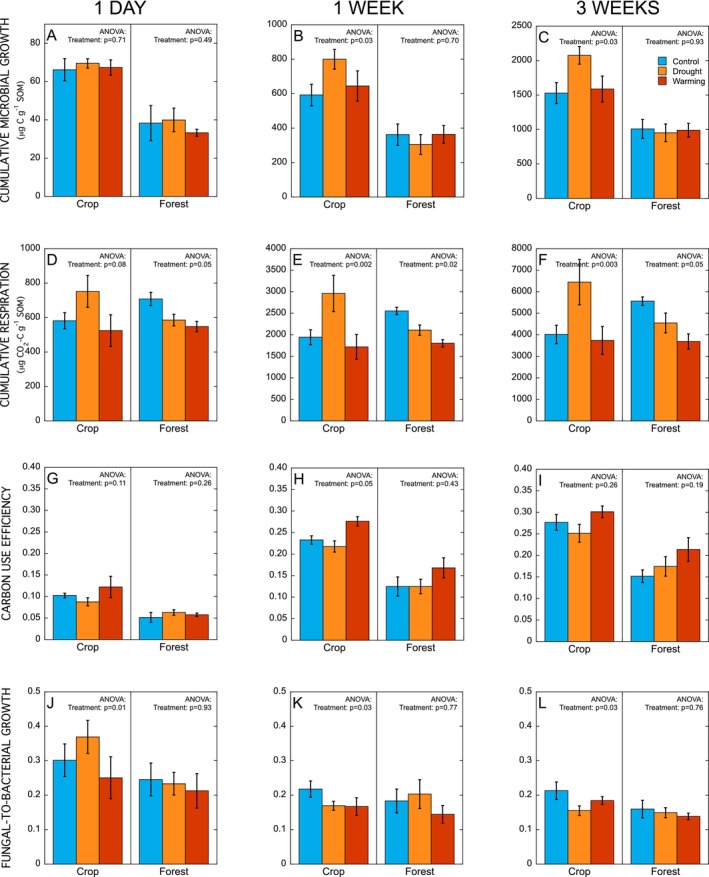
Cumulative microbial growth (Panels A–C), respiration (Panels D–F), carbon use efficiency (Panels G–I), and the ratio of fungal‐to‐bacterial growth (Panels J–L) in cropland and forest soils during 1 day, 1 week, and 3 weeks after drying‐rewetting. Data represent mean ± SE (*n* = 4 for control and drought treatments and *n* = 3 for warming treatment).

In the cropland soil, the field treatments had a significant effect on cumulative microbial growth, with cumulative growth 1 week (*p* = 0.03) and 3 weeks (*p* = 0.03) after rewetting being higher in the drought treatment than the control, with the warming treatment intermediate (Figure 5B,C). Cumulative respiration was also higher in soils from the drought treatment, both during 1 week (*p* = 0.002) and 3 weeks (*p* = 0.003) after rewetting (Figure 5E,F), and consequently there was no significant effect of the field treatments on microbial CUE after rewetting (Figure 5D–F). However, the ratio of fungal‐to‐bacterial growth after rewetting did vary among field treatments (Figure 5J–L). During the first day after rewetting, the ratio of fungal‐to‐bacterial growth was higher in soil from the drought treatment (*p* = 0.01), but this response changed over time, as after 1 and 3 weeks after rewetting, the overall ratio of fungal‐to‐bacterial growth was lower in the drought treatment than the control (*p* = 0.03). These dynamics induced by rewetting therefore contrasted with the ratio of fungal‐to‐bacterial growth at steady‐state, which did not vary among fieldtreatments (*p* = 0.21).

In contrast with the cropland soils, the field treatments had no significant effect on cumulative microbial growth in the forest soils after rewetting (all *p* > 0.49; Figure 5A–C). Cumulative respiration during 1 day (*p* = 0.05), 1 week (*p* = 0.02), and 3 weeks (p = 0.05) after rewetting was lower in soils from the drought and warming treatments than the control (Figure 5D–F). However, there was no significant effect of the fieldtreatments on microbial CUE after rewetting (*p* > 0.19; Figure 5G–I). There was also no significant effect of field treatments on the ratio of fungal‐to‐bacterial growth after rewetting in the forest soils (all *p* > 0.76; Figure 5J–L).

### 3.3 Effect of Drying‐Rewetting on Microbial Community Structures

**FIGURE 6:**
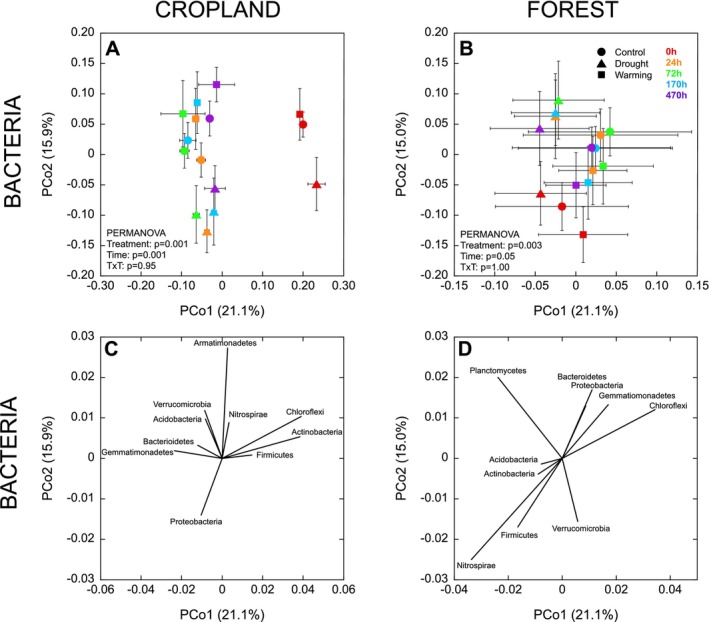
Changes in bacterial community structure (beta diversity) over time following drying‐rewetting. Panels C and D show bacterial phyla explaining changes in community structure shown in Panels A and B, respectively. Data represent mean ± SE (*n* = 4 for control and drought treatments and *n* = 3 for warming treatment). Different symbols denote field treatments and different colors denote time points.

**FIGURE 7:**
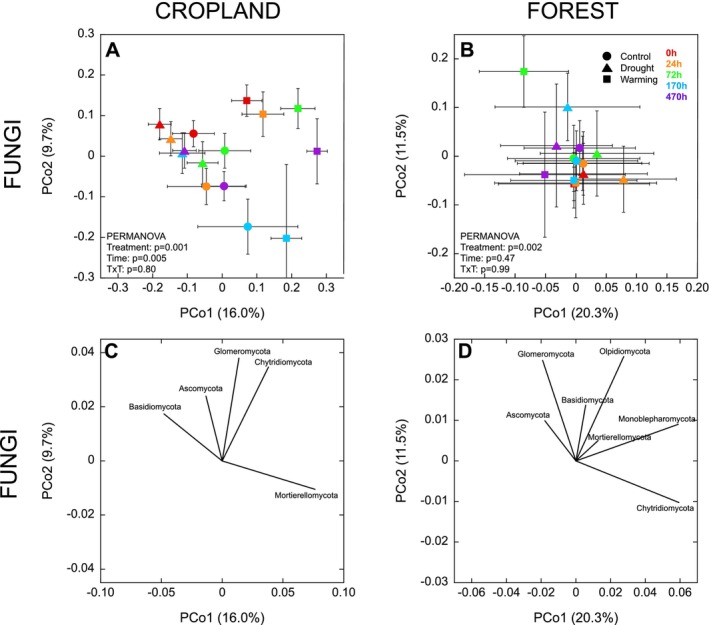
Changes in fungal community structure (beta diversity) over time following drying‐rewetting. Panels C and D show fungal phyla explaining changes in community structure shown in Panels A and B, respectively. Data represent mean ± SE (*n* = 4 for control and drought treatments and *n* = 3 for warming treatment). Different symbols denote field treatments and different colors denote time points.

In the cropland, there was a significant effect of field treatment and time on both bacterial (Figure 6A) and fungal community structures (Figure 7A) after rewetting. In contrast, in the forest, the microbial community was less responsive to the disturbance, with only a marginal effect of time on the bacterial community structure (*p* = 0.05; Figure 6B), and no effect of time on the fungal community structure (*p* = 0.47; Figure 7B). In the cropland, for bacteria, there was clear separation between 0 h and the other time points, but also a more subtle trajectory of change between 24 and 470 h, where the bacterial community initially became more different from the 0 h community at 24 and 72 h, before gradually converging back towards the 0 h community at 170 and 470 h (Figure 6A). These differences in bacterial community structure were associated with a higher relative abundance of Actinobacteria, Chloroflexi, and Firmicutes at 0 h, and a higher relative abundance of Gemmatimonadetes at 24 and 72 h after rewetting (Figure 6C). Although less pronounced, a similar tendency for a trajectory of change could be seen for the bacterial community in the forest soils, as the 0 h community tended to separate most strongly from the 24 and 72 h communities, before the communities at 170 and 470 h became more similar to that at 0 h. In this case, bacterial community structures at 0 h were associated with a higher relative abundance of Actinobacteria, Firmicutes, Verrucomicrobia, and Nitrospirae, compared to 24 and 72 h after rewetting, where there was a higher relative abundance of phyla including Bacteroidetes and Proteobacteria and Gemmatimonadetes (Figure 6D).

For fungi, in the cropland, the difference between the communities at 0 h and after rewetting was less pronounced than for bacteria, with small differences between community structures at 0, 24 and 72 h after rewetting and a larger shift in community structure at 170 h after rewetting (Figure 7A). The initial fungal community at 0 h was associated with a higher relative abundance of Glomeromycota, while at 170 h there was a higher relative abundance of Mortierellomycota (Figure 7C).

### 3.4 Linking Microbial Community Structure and Function

There was no significant relationship between initial microbial community structure and recovery times for bacterial and fungal growth in either the cropland (all *p* > 0.30) or forest (all *p* > 0.27) soils. In the cropland soils, there was a significant negative relationship between cumulative fungal growth after rewetting and the PCo2 score describing differences in fungal community structure (*R* ^2^ = 0.08, *p* = 0.04), as well as a trend for a negative relationship between cumulative bacterial growth after rewetting and the PCo1 score describing differences in bacterial community structure (*R* ^2^ = 0.06, *p* = 0.07). There were no significant relationships between cumulative microbial growth and microbial community structures in the forest (all *p* > 0.41).

## 4 Discussion

### 4.1 Field Treatment Effects on Initial Soil and Microbial Characteristics

The aim of this study was to investigate how legacies of drought and warming affected microbial functional and structural responses to an experimental drought cycle in tropical cropland and forest soils. Our experimental treatments of rain‐exclusion shelters and OTCs successfully simulated the effects of climate change by reducing soil moisture in both landuses (Figure 1A,B) and by increasing soil temperature in the cropland (Figure 1C). Under the canopy, in the forest, the installed OTCs had no clear effect on soil temperature (Figure 1D) or moisture (Figure 1B). Therefore, discussion on the effects of simulated climate warming in the forest is precluded.

The field treatments altered microbial community structures (Figure 2) but did not generally induce changes in physicochemical characteristics, including SOM and EC (Table 1), suggesting that microbial communities are faster to respond to environmental perturbations than soil physicochemistry. In the cropland, soil pH was lower in the drought treatment, and the bacterial community also diverged from the control (Figure 2C). Although pH is known to have a strong influence on bacterial community structure, rather minimal effects would be expected within the pH range of 6.4 (in the drought treatment) to 6.8 (in the control treatment), with pH changes below 6.0 found to induce the most pronounced community shifts (Rousk et al. 2010). Instead, the differences that we observe are in line with studies that have linked environmental changes in soil moisture to bacterial community shifts. For example, several studies have found that Planctomycetes (often considered slow‐growing *K*‐strategists) and Deinococcus‐Thermus (extremophiles) may be favored by drought (Bouskill et al. 2013; Chodak et al. 2015; Drigo et al. 2017). The field treatments also restructured the fungal community in the cropland, leading to differences between the warming and drought treatments, with the control intermediate (Figure 2D). This suggested a divergence of the fungal community in response to simulated drought (affecting soil moisture) and warming (affecting both soil temperature and moisture). Although fungi are thought to be more tolerant than bacteria to low moisture (Yuste et al. 2011; de Vries et al. 2018; Leizeaga et al. 2021), several studies have identified a high sensitivity of fungal community composition to drought (Bastida et al. 2017; Sayer et al. 2017). Our results also show that in the studied tropical cropland soils, up to 10 vol% lower soil moisture was sufficient to drive changes in fungal community structure. In contrast, despite a reduction of up to 25 vol% soil moisture during the wet period in the forest, the drought treatment had no significant effect on bacterial or fungal community structures (Figure 2E,F). This may be explained by seasonality at the site, with soil moisture reaching much lower levels during the dry period, irrespective of field treatments (Figure 2B). These periods of low moisture may provide a stronger filter for microbial selection (also see Section 4 below), making any more subtle changes in microbial structure induced by the field treatments harder to identify.

### 4.2 Climate‐Legacy Effects on Microbial Functional Responses to Drying‐Rewetting

To assess the legacy effect of field treatments, all soils were subjected to a standardized perturbation, in order to identify how microbial community traits (“resilience”) had been shaped by the different climate legacies. In the cropland, both the drought and warming field treatments reduced soil moisture, with a similar effect size (Figure 1A), while only the drought treatment reduced soil moisture in the forest (Figure 1B). If soil moisture was a driver for microbial drought resilience, we would expect an enhanced resilience in soils from both field‐treatments in the cropland, while only the drought treatment would affect microbial resilience in the forest. Contrary to this, microbial growth started increasing immediately in all soils, irrespective of field treatments (Figure 3A,B), matching responses previously assessed in drylands (Leizeaga et al. 2021), and indicating a generally high resilience to drought. This suggests that the microbial communities in all soils had been exposed to this type of disturbance before and thus did not perceive the event as “harsh” (Meisner et al. 2017). In support of this, studies assessing the spatial and temporal extent of drought in the Ethiopian highlands have shown recurrent cycles of drought ranging from mild to severe during the last decades (Mekonen, Berlie, and Ferede 2020; Alito and Kerebih 2024).

In the cropland, microbial growth and respiration were both more responsive in soils with a legacy of drought, reaching higher rates after rewetting (Figure 3A,D). This could be explained by higher resource availability in these soils (see below), which could support higher metabolic rates. However, despite differences in microbial process rates, this did not translate into differences in microbial CUE (Figure 3G), suggesting that the capacity of microorganisms to assimilate C after drought disturbances did not depend on the short‐term climate history. A similar response was found in a mountain grassland, where coupled responses in growth and respiration after drying‐rewetting resulted in no legacy effect of one‐year drought on CUE, while there was evidence for a drought legacy effect on CUE after 10 years (Canarini et al. 2021). As such, longer‐lasting changes in climate that repeat year after year could compound to result in more pronounced legacy effects on microbial functions than are evident in the short term.

Steady‐state microbial growth and respiration rates were higher in cropland soils with a legacy of drought (Figure 3C,F), which contrasts with the expectation that drought would reduce plant‐derived C inputs to soil (Ruehr et al. 2009; Fuchslueger et al. 2014), leading to lower microbial process rates (Hicks et al. 2018). One explanation for this surprising finding may be that even if the quantity of plant‐derived C inputs decreases with drought, the quality of inputs may increase, as recent studies have identified changes in the chemical composition of root exudates during drought that might support higher microbial activities (Canarini, Merchant, and Dijkstra 2016; Gargallo‐Garriga et al. 2018; de Vries et al. 2019). Alternatively, lower levels of soil moisture as a result of the drought field treatment may have suppressed the decomposition of plant residues and SOM in the field, leaving a larger pool of C (Table 1) that was more susceptible to decomposition upon rewetting, or there may have been a build‐up of labile C substrate due to continued depolymerization of organic matter during dry periods, as enzymes require thinner water films than larger microbial cells, meaning that depolymerization by extracellular enzymes continues after microbial metabolism stops (Schimel 2018). This may have resulted in an accumulation of more labile C and thus higher resource availability when substrate diffusion resumed upon rewetting (Geisseler, Horwath, and Scow 2011; Schaeffer et al. 2017). Interestingly, although both the drought and warming treatments in the cropland led to a similar reduction in soil moisture, microbial growth and respiration in the soils with a legacy of warming were not enhanced like we saw for the soils with a legacy of drought. This may be because decomposition in these soils was accelerated by higher temperatures, leading to substrate depletion (Melillo et al. 2002), which counteracted any potential increase in microbial resource availability arising from lower levels of moisture.

Although the legacy of drought did not affect the type of microbial growth response to the experimental drought cycle (Figure 5A,B), there was a tendency for a faster recovery of fungal growth in soils with a legacy of drought, with this trend seen for both the cropland and forest (Figure 4B). In the case of the cropland soils, this also manifested as a higher ratio of fungal‐to‐bacterial growth—and thus an initial period of more fungal‐dominated decomposition—in soil from the drought treatment during the first day after rewetting (Figure 5J). This suggests that fungi in soils with a history of drought were poised to recover their growth rates more quickly, perhaps as a result of being previously exposed to more drought‐cycles in the field, resulting in selection for a more drought‐tolerant fungal community (de Vries et al. 2018). Interestingly, in both land uses, fungi recovered more quickly than bacteria (Figure 4). Our finding therefore contrasts with previous findings that fungi are more resistant but less resilient to drought than bacteria (Barnard, Osborne, and Firestone 2013; de Vries and Shade 2013), by showing that fungi can also demonstrate high resilience to drought.

Given the differences in moisture reduction induced by the rain shelters in the forest (up to 25 vol% lower moisture) and cropland (up to 10 vol% lower moisture), we expected that the drought‐legacy effects would be more pronounced in the forest than cropland soils. However, the effects on soil moisture were primarily observed during the wet period, and moisture was consistently low in soils from all field treatments during the dry period. Rather than the average soil moisture, it is likely that it is the period of low moisture in particular that selects for a microbial community with traits conferring resistance and resilience to drought. This is because microbial physiology and activity are most strongly affected by low water potential (Manzoni, Schimel, and Porporato 2012). While soils are wet, changes in moisture can occur without large reductions in water potential, whereas when soils are dry, relatively small reductions in water content can generate large changes in water potential (Schimel 2018). Indeed, seasonal variability in moisture was found to drive larger differences in microbial community composition than experimental precipitation manipulation treatments in a semiarid woodland (Cregger et al. 2012). A recent study of more humid ecosystems also suggested that there may be a general threshold for detecting effects of reduced precipitation on microbial resilience to drought, finding that > 30% reduction in precipitation was required to induce detectable changes at typical levels of experimental replication (Tang et al. 2023). As such, the differences in soil moisture (< 25 vol%) induced by the field treatments during the wet period may have been a relatively weak selective pressure, explaining why we do not see more pronounced effects of the rain‐exclusion treatments on microbial responses here.

### 4.3 Land‐Use Effects on Microbial Responses to Drying‐Rewetting

We hypothesized that microbial responses to the experimental drought cycle would be more resilient and C‐use efficient in the cropland than in forest soils. Our expectation for a history of higher exposure to drought disturbances in the croplands could be validated as soil moisture during the dry season was 33% lower in the cropland compared to the forest (ca. 8 vol% vs. 12 vol%; Figure 1A,B). Cropland and forest soils both exhibited resilient responses to the experimental drought cycle with immediate increases in growth rate (Figure 3A,B), suggesting that the microbial communities in soils from both land uses experienced the perturbation as relatively mild (Meisner et al. 2017). However, while the type of growth response to rewetting did not categorically differ between land uses, the rate of microbial growth after rewetting was generally higher in the cropland than forest soils (Figure 3A,B), despite respiration rates being very similar (Figure 3D–F). Consequently, microbial CUE after rewetting was typically higher in the cropland than in forest soils (Figure 3G,H). This suggested that the observed higher CUE after rewetting in drier sites along natural precipitation gradients (Leizeaga et al. 2021; Tang et al. 2023) could be extended to also include land‐use‐generated differences in soil moisture, where there may be a higher potential for microbial assimilation of C in cropland than forest soils in response to future cycles of drought.

There was no clear difference between land uses in the time for bacterial and fungal recovery after rewetting (Figure 4). However, microbial growth in the cropland soils started from a higher rate and was also more responsive after rewetting, with rates reaching a peak and then declining (Figure 5A) compared to the forest, where rates simply stabilized (Figure 5B). This suggests subtle differences in microbial community traits between landuses. It has been suggested that the SOM in croplands is of a higher quality (as defined by a lower C/N ratio and higher microbial biomass per unit SOM; Woloszczyk et al. 2020). Using microbial C use (growth + respiration) per unit SOM as an index for SOM quality, reflecting the microbial assimilability (Xu et al. 2014), we also found support for a higher quality SOM in the cropland than forest (12.5 ± 1.1 and 10.4 ± 0.8 μg C g^−1^ SOM h^−1^, respectively). As such, the higher quality SOM available in the cropland may have been invested in drought resistance traits (Malik and Bouskill 2022), potentially explaining why microbial growth could start from a higher level immediately after rewetting. Added to this, a history of repeated physical disturbances from ploughing in the studied site may also have selected for a microbial community poised for fast colonization (Philippot, Griffiths, and Langenheder 2021; Hicks, Lin, and Rousk 2022), which could further add to the pronounced growth dynamics in the cropland soils after rewetting (also see below).

### 4.4 Structural Microbial Responses to Drying‐Rewetting

We observed pronounced shifts in microbial community structures after rewetting, with community structures in the cropland soils being particularly responsive (Figures 6A and 7A), in line with the more pronounced microbial growth responses there (Figure 3A). Bacterial communities underwent a pronounced change between 0 h and 24 h after rewetting, with the community continuing to diverge from the initial community at 72 and 170 h, before then starting to return towards the initial community at 470 h (Figure 6A,B). This trajectory of change occurred in both the cropland and forest soils, suggesting an ordered succession, although the shifts were associated with changes in different taxa in the different land uses (Figure 6C,D). Actinobacteria were abundant in the air‐dried soils from both land uses, consistent with being identified as good drought‐resistors due to osmolyte production (Goodfellow and Williams 1983; Zvyagintsev et al. 2007; Barnard, Osborne, and Firestone 2013). Proteobacteria have often been found to exhibit strong responses to rewetting (Blazewicz et al. 2020; Placella, Brodie, and Firestone 2012; Engelhardt et al. 2018), as we also found in our forest soils. In contrast, Gemmatimonadetes were previously identified as exhibiting a more stable and resistant life strategy, being relatively insensitive to both dry‐down and wet‐up (Barnard, Osborne, and Firestone 2013), whereas our results from the cropland soil suggest that taxa from this phylum responded quickly to rewetting, increasing in relative abundance within 24 h.

Several studies have suggested that fungi may be more tolerant to drying than bacteria (de Vries et al. 2018; Leizeaga et al. 2021), potentially resulting in less extreme changes in fungal than bacterial community structures after drought (Barnard, Osborne, and Firestone 2013). Consistent with these reports, the fungal community was generally less responsive after the experimental drought cycle, with no or very slow responses after rewetting (Figure 7A,B), therefore contrasting with the immediate shift seen in the bacterial community structure (Figure 6A). In the cropland, the shift in the fungal community was associated with a higher relative abundance of Glomeromycota at 0 h, and a higher relative abundance of Mortierellomycota at 170 h (Figure 7C). As Glomeromycota are arbuscular mycorrhizal fungi (Redecker and Raab 2006), the high relative abundance observed before rewetting was likely a remnant from the initial soil system, with this group unable to respond after the disturbance due to the removal of host plants in our study. In contrast, Mortierellomycota—dominated by Mortierella—are known to be fast‐growing saprotrophs, able to respond quickly to changes in the environment, and particularly associated with the colonization of new labile sources of organic matter (Li et al. 2018; Maillard et al. 2020; Fracchia et al. 2021). These traits suggest that Mortierellomycota may have been able to recolonize the new sources of organic matter released by rewetting more quickly than other fungal taxa, resulting in the increased relative abundance by 170 h that we observed.

## 5 Conclusion

We assessed how climate legacies affected microbial functional and structural responses after drought in tropical cropland and forest soils. Microbial growth started increasing immediately in all soils after rewetting, with this resilient response suggesting that microbial communities were well disposed to this type of disturbance. Simulated drought and warming led to pronounced shifts in bacterial and fungal community structures in the cropland that were also responsive during the experimental drought cycle. Microbial communities were also more functionally responsive after rewetting in the cropland soils, demonstrating a more metabolically active and efficient microbial community responding to disturbances there. A legacy of drought particularly enhanced microbial growth and respiration in the cropland but not in the forest. Together, these results suggest contrasting feedbacks to climate change determined by land use. Although the conversion from pristine forest to cropland causes soil C loss, the remaining C may be less vulnerable to drought cycles. Cropland soils were associated with a higher microbial assimilation of C after rewetting, which may mitigate C losses in responses to future cycles of drought, compared to forests where soil C reservoirs remain more sensitive.

## Author Contributions

**Lettice C. Hicks:** conceptualization, data curation, formal analysis, investigation, methodology, visualization, writing – original draft, writing – review and editing. **Johannes Rousk:** conceptualization, funding acquisition, investigation, methodology, project administration, resources, supervision, writing – review and editing. **Hans Sandén:** conceptualization, funding acquisition, investigation, project administration, supervision, writing – review and editing. **Menale Wondie:** conceptualization, funding acquisition, investigation, methodology, project administration, resources, supervision, writing – review and editing. **Carla Cruz Paredes:** data curation, formal analysis, investigation, visualization, writing – review and editing. **Ainara Leizeaga:** conceptualization, data curation, formal analysis, investigation, methodology, visualization, writing – original draft, writing – review and editing. **Dániel Tájmel:** investigation, writing – review and editing. **Albert C. Brangarí:** investigation, writing – review and editing.

## Conflicts of Interest

The authors declare no conflicts of interest.

## Supporting information


Figures S1–S2

## Data Availability

The data that support the findings of this study are openly available in Zenodo at https://doi.org/10.5281/zenodo.14679177. The sequence data are available from National Center for Biotechnology Information at https://www.ncbi.nlm.nih.gov/bioproject/PRJNA1144366.
