# Supplementary material for: Simulated Climate Change Enhances Microbial Drought Resilience in Ethiopian Croplands but Not Forests
Source: Glob Chang Biol. 2025 Mar 5;31(3):e70065. doi: 10.1111/gcb.70065 (PMC11881793; doi:10.1111/gcb.70065)
Supplement: Supplementary file 1 — Figures S1–S2 [file GCB-31-e70065-s001.docx]

**Supporting Information**

**Simulated climate change enhances microbial drought resilience in Ethiopian croplands but not forests**

Lettice C Hicks, Ainara Leizeaga, Carla Cruz-Paredes, Albert C Brangarí, Dániel Tájmel, Menale Wondie, Hans Sandén, Johannes Rousk

**Figure S1** Schematic diagram of conducted experiment. Soils from contrasting land-uses (tropical cropland and forest) including climate change simulation treatments at both sites (control, rain-shelters simulating drought and open-top chambers simulating warming) were all exposed to a standardized dry-wet cycle.

** Figure S2** Phyla explaining variation in initial bacterial (Panels A and C) and fungal (Panels B and D) community structures in cropland and forest soils (shown in Fig. 2).
